# High-Intensity Interval Training for Individuals With Isolated Impaired Fasting Glucose: Protocol for a Proof-of-Concept Randomized Controlled Trial

**DOI:** 10.2196/59842

**Published:** 2025-02-20

**Authors:** Sathish Thirunavukkarasu, Thomas R Ziegler, Mary Beth Weber, Lisa Staimez, Felipe Lobelo, Mindy L Millard-Stafford, Michael D Schmidt, Aravind Venkatachalam, Ram Bajpai, Farah El Fil, Maria Prokou, Siya Kumar, Robyn J Tapp, Jonathan E Shaw, Francisco J Pasquel, Joe R Nocera

**Affiliations:** 1 Department of Family and Preventive Medicine School of Medicine Emory University Atlanta, GA United States; 2 Emory Global Diabetes Research Center Woodruff Health Sciences Center Emory University Atlanta, GA United States; 3 Division of Endocrinology, Metabolism and Lipids Department of Medicine Emory University School of Medicine Atlanta, GA United States; 4 Hubert Department of Global Health Rollins School of Public Health Emory University Atlanta, GA United States; 5 Exercise Physiology Laboratory School of Biological Sciences Georgia Institute of Technology Atlanta, GA United States; 6 Department of Kinesiology University of Georgia Atlanta, GA United States; 7 Oxford College Emory University Atlanta, GA United States; 8 School of Medicine Keele University Staffordshire United Kingdom; 9 University of Nicosia Medical School Nicosia Cyprus; 10 College of Sciences Georgia Institute of Technology Atlanta, GA United States; 11 Research Institute for Health and Wellbeing Coventry University Coventry United Kingdom; 12 Baker Heart and Diabetes Institute Melbourne Australia; 13 Department of Medicine School of Medicine Emory University Atlanta, GA United States; 14 Division of Physical Therapy, Departments of Neurology and Rehabilitation Medicine School of Medicine Emory University Atlanta, GA United States; 15 Center for Visual and Neurocognitive Rehabilitation Atlanta, GA United States

**Keywords:** isolated impaired fasting glucose, prediabetes, high-intensity interval training, fasting hyperglycemia, diabetes incidence, fasting glucose, glucose, diabetes, proof of concept, interval training, type 2 diabetes, hyperglycemia, overweight, obese, weight, insulin, feasibility

## Abstract

**Background:**

Standard lifestyle interventions have shown limited efficacy in preventing type 2 diabetes among individuals with isolated impaired fasting glucose (i-IFG). Hence, tailored intervention approaches are necessary for this high-risk group.

**Objective:**

This study aims to (1) assess the feasibility of conducting a high-intensity interval training (HIIT) study and the intervention acceptability among individuals with i-IFG, and (2) investigate the preliminary efficacy of HIIT in reducing fasting plasma glucose levels and addressing the underlying pathophysiology of i-IFG.

**Methods:**

This study is a 1:1 proof-of-concept randomized controlled trial involving 34 physically inactive individuals (aged 35-65 years) who are overweight or obese and have i-IFG. Individuals will undergo a 3-step screening procedure to determine their eligibility: step 1 involves obtaining clinical information from electronic health records, step 2 consists of completing questionnaires, and step 3 includes blood tests. All participants will be fitted with continuous glucose monitoring devices for approximately 80 days, including 10 days prior to the intervention, the 8-week intervention period, and 10 days following the intervention. Intervention participants will engage in supervised HIIT sessions using stationary “spin” cycle ergometers in groups of 5 or fewer. The intervention will take place 3 times a week for 8 weeks at the Aerobic Exercise Laboratory in the Rehabilitation Hospital at Emory University. Control participants will be instructed to refrain from engaging in intense physical activities during the study period. All participants will receive instructions to maintain a eucaloric diet throughout the study. Baseline and 8-week assessments will include measurements of weight, blood pressure, body composition, waist and hip circumferences, as well as levels of fasting plasma glucose, 2-hour plasma glucose, and fasting insulin. Primary outcomes include feasibility parameters, intervention acceptability, and participants’ experiences, perceptions, and satisfaction with the HIIT intervention, as well as facilitators and barriers to participation. Secondary outcomes comprise between-group differences in changes in clinical measures and continuous glucose monitoring metrics from baseline to 8 weeks. Quantitative data analysis will include descriptive statistics, correlation, and regression analyses. Qualitative data will be analyzed using framework-driven and thematic analyses.

**Results:**

Recruitment for the study is scheduled to begin in February 2025, with follow-up expected to be completed by the end of September 2025. We plan to publish the study findings by the end of 2025.

**Conclusions:**

The study findings are expected to guide the design and execution of an adequately powered randomized controlled trial for evaluating HIIT efficacy in preventing type 2 diabetes among individuals with i-IFG.

**Trial Registration:**

Clinicaltrials.gov NCT06143345; https://clinicaltrials.gov/study/NCT06143345

**International Registered Report Identifier (IRRID):**

PRR1-10.2196/59842

## Introduction

The prevalence of type 2 diabetes is increasing globally [1-3], driven predominantly by a rising number of individuals with prediabetes [2]. Globally, an estimated 860 million (8.4%) adults are living with prediabetes, a condition that increases the risk of developing type 2 diabetes [2], micro- and macrovascular complications, and mortality [4].

Prediabetes is not a singular entity but rather a heterogeneous group of metabolic defects that often precede type 2 diabetes [5-7]. Prediabetes phenotypes include isolated impaired fasting glucose (i-IFG), isolated impaired glucose tolerance (i-IGT), and IFG + IGT. Each prediabetes phenotype exhibits distinct pathophysiological abnormalities [5-7]. i-IFG is marked by impaired early-phase insulin secretion and hepatic insulin resistance. Conversely, i-IGT involves impairments in both early- and late-phase insulin secretion and skeletal muscle insulin resistance [5,7]. IFG + IGT presents a combination of defects observed in both i-IFG and i-IGT [5,7]. i-IFG accounts for a substantial portion of the global prediabetes population, ranging from 43.9% to 58% among Caucasian individuals and 29.2% to 48.1% among Asian individuals, depending on the diagnostic criteria [8]. Individuals with i-IFG exhibit a 4 to 5.5 times higher rate of progression to type 2 diabetes, depending on the diagnostic criteria, compared to those with normoglycemia [9].

Individuals with prediabetes are typically advised to adopt standard lifestyle interventions that emphasize improving diet quality with a modest calorie restriction and increasing moderate-intensity physical activity to reduce the risk of developing type 2 diabetes [10,11]. However, recent research highlights the varied effectiveness of these interventions among different prediabetes phenotypes. While these approaches prove highly effective for individuals with i-IGT and IFG plus IGT, their efficacy is notably limited for those with i-IFG [6,12,13]. Thus, there arises a necessity for alternative lifestyle intervention strategies tailored specifically to individuals with i-IFG.

One of the promising approaches is high-intensity interval training (HIIT), recognized as a time-efficient exercise option with significant benefits for metabolic health [14]. HIIT entails alternating short bursts of high-intensity exercise with periods of less active or passive recovery [15]. It is noteworthy that HIIT represents a more intensive exercise regimen compared to the current physical activity recommendations for individuals with prediabetes [10,11].

**Figure 1:**
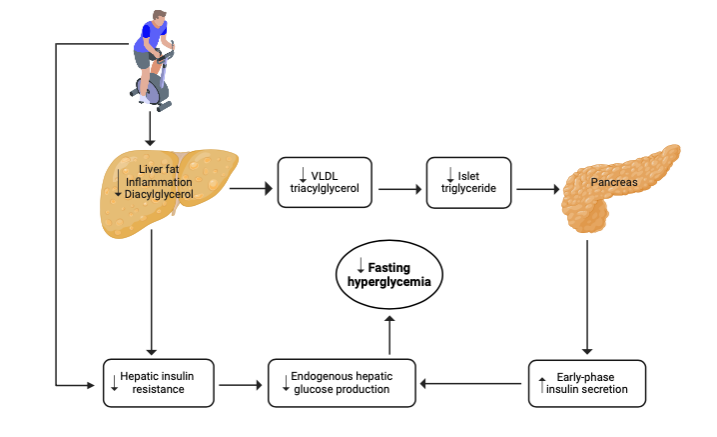
Potential pathways through which high-intensity interval training sessions may address the pathophysiological abnormalities and fasting hyperglycemia in individuals with isolated impaired fasting glucose. VLDL: very low-density lipoprotein.

HIIT has been shown to effectively reduce hepatic insulin resistance and improve early-phase insulin secretion in individuals with type 2 diabetes [16-20], leading to significant reductions in fasting plasma glucose (FPG) levels [17,21-25]. Given that i-IFG shares these same pathophysiological defects [5-7], it is reasonable to hypothesize that HIIT could also be effective in individuals with i-IFG, as depicted in Figure 1. However, this hypothesis has yet to be tested in a randomized controlled trial (RCT). This is a critical investigation, as reducing fasting hyperglycemia is key to preventing the progression of type 2 diabetes in those with i-IFG [6,26]. To inform the design and implementation of this RCT, we propose conducting a proof-of-concept study among individuals with i-IFG, with the following objectives.

- Primary objectives (feasibility and acceptability): (1) assess the feasibility of recruiting and retaining participants and executing study procedures; (2) examine the feasibility, acceptability, and appropriateness of the HIIT intervention for participants; and (3) investigate participants’ experiences, perceptions, and satisfaction with the HIIT intervention, and identify facilitators and barriers to participation.
- Secondary objective (preliminary efficacy): Investigate the preliminary efficacy of HIIT in reducing FPG levels and addressing the underlying pathophysiology of i-IFG.

## Methods

### Study Design, Study Setting, and Participants

**Figure 2:**
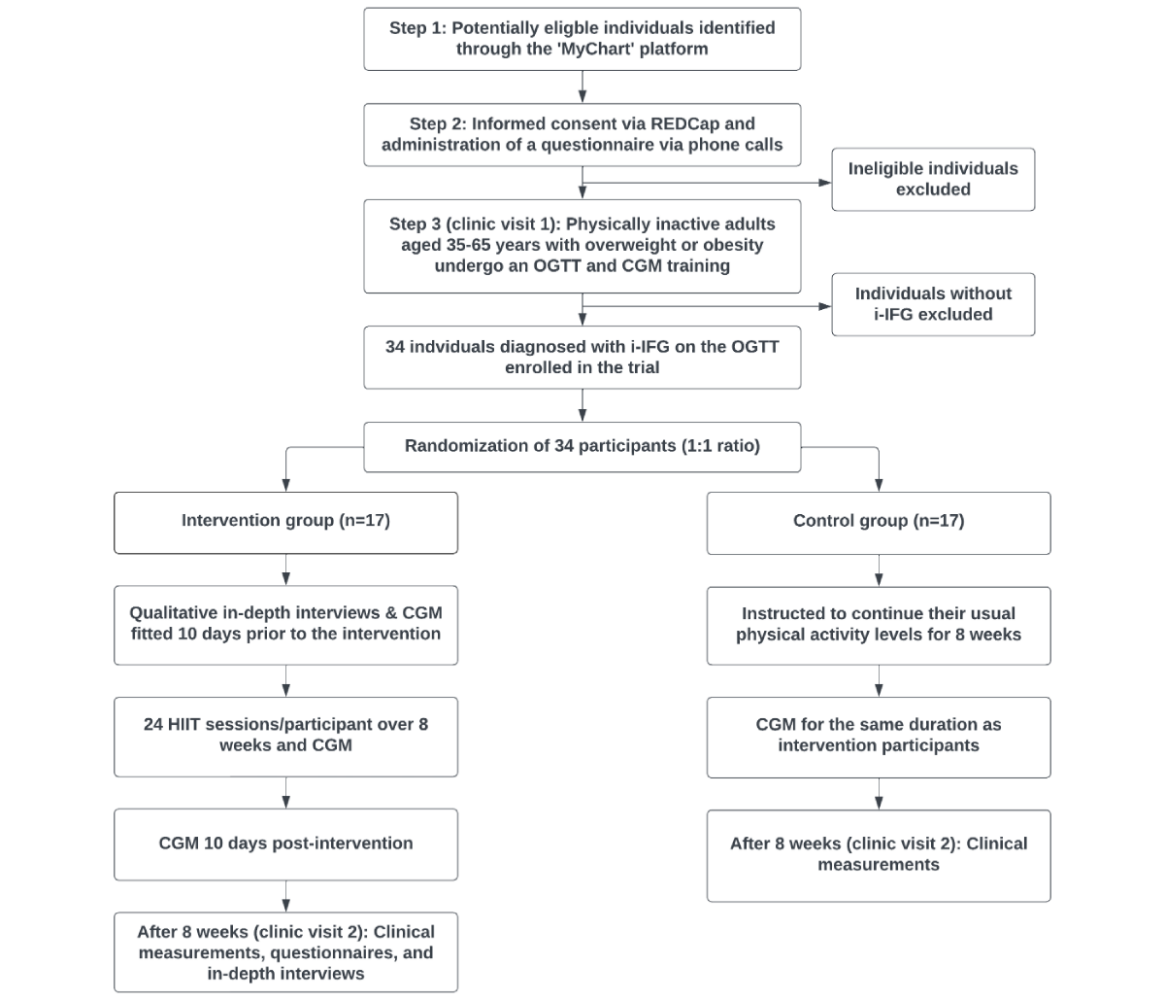
CONSORT (Consolidated Standards of Reporting Trials) flow diagram. CGM: continuous glucose monitoring; HIIT: high-intensity interval training; i-IFG: isolated impaired fasting glucose; OGTT: oral glucose tolerance test; REDCap: Research Electronic Data Capture.

The study will be reported in accordance with the CONSORT (Consolidated Standards of Reporting Trials) guidelines for randomized pilot and feasibility trials [27]. This is a “proof-of-concept” 1:1 parallel-group RCT involving 34 physically inactive individuals aged 35-65 years who are overweight or obese and have i-IFG. Figure 2 presents the study’s CONSORT diagram. The Georgia Clinical Research Center (GCRC) at Emory University Hospital will serve as the site for participant recruitment and conducting study procedures. A highly trained and experienced study coordinator will recruit participants through a comprehensive 3-step screening procedure.

### Step 1: Screening (via Electronic Health Records)

Potential participants will be identified using Emory’s electronic health care records system, known as “MyChart.” Queries within this database will target individuals aged 35-65 years with a BMI≥25 kg/m^2^ (≥23 kg/m^2^ if Asian descent) [28], who have been diagnosed with prediabetes (hemoglobin A_1c_ [HbA_1c_] 5.7%-6.4%) [29] within the last 12 months, have no diagnosis of diabetes (FPG≥126 mg/dL or 2-hour plasma glucose ≥200 mg/dL or HbA_1c_≥6.5% or currently taking antidiabetic drugs) [29], not currently taking weight-loss medications, not currently taking drugs known to influence glucose tolerance (steroids and antipsychotics), not currently taking beta-blockers and calcium channel blockers (individuals taking these drugs will not reach heart rate (HR) targets for the HIIT sessions), did not undergo bariatric surgery, no anemia (anemia may limit the exercising capacity), and have no chronic illnesses (cardiovascular disease, stroke, cancers, chronic respiratory diseases, and mental health disorders). Individuals meeting these criteria will receive an invitation through the MyChart platform to participate in the second screening step.

### Step 2: Screening (via Phone Calls)

Those expressing interest in participating in the study through MyChart will be contacted via phone. During these calls, individuals will receive a comprehensive explanation of the study and have any questions addressed. They will then be asked to sign an electronic consent form via Emory’s REDCap (Research Electronic Data Capture; Vanderbilt University) platform [30]. Following consent, potential participants will complete a questionnaire to assess their eligibility for step 3 screening based on the following criteria.

- Physically inactive (less than 150 minutes per week of moderate-intensity physical activity, less than 75 minutes per week of vigorous-intensity physical activity, and <600 metabolic equivalent task–minutes per week) [31] assessed by the International Physical Activity Questionnaire (IPAQ) [32].
- Available to participate in the HIIT group sessions.
- No history of smoking (smoking is associated with reduced insulin secretion and increased insulin resistance) [33].
- Not enrolled in weight loss programs in the past 6 months.
- Not enrolled in any regular exercise programs in the past 6 months.
- Not currently following a specific diet (eg, ketogenic and Mediterranean).
- No plans to undergo bariatric surgery during the study period.
- No plans to relocate outside the study area during the study period.
- Not pregnant.
- Not breastfeeding.

### Step 3: Screening (via In-Person Clinic Visits)

Individuals meeting the step 2 criteria will be invited to visit the GCRC at Emory University Hospital after fasting overnight for a minimum of 8 hours [29]. During the visit, participants will complete standard questionnaires to collect sociodemographic information (education, occupation, and marital status) [34,35] and details on alcohol consumption [34,35] and dietary intake [36]. Additionally, physical measurements will be conducted using standardized instruments in accordance with the World Health Organization’s STEPwise approach to noncommunicable disease risk factor surveillance (STEPS) protocol [37]. Following these assessments, individuals will undergo an oral glucose tolerance test (OGTT) and provide blood samples for insulin. Individuals diagnosed with i-IFG, defined by the American Diabetes Association criteria as FPG between 100-125 mg/dL and 2-hour plasma glucose<140 mg/dL [29], will be deemed eligible to participate in the study. Individuals without i-IFG will be excluded from further participation in the study. They will receive a summary report of their test results and general healthy lifestyle advice and will be referred to their general practitioner if they have IGT or undiagnosed diabetes for further management.

### Randomization and Blinding

Participants will be equally randomized into either the intervention or control group after completing baseline assessments and being found eligible, using a computer-generated randomization sequence by a statistician not involved in the trial. Given the nature of the study, only specific personnel such as nursing staff, laboratory personnel, and the data analyst will be blinded to participant allocation to the study groups. Participants, the study coordinator, the HIIT intervention instructor, and the principal investigator will not be blinded to participation allocation.

### Intervention

Following the recommendation of the American College of Sports Medicine [38], participants in the intervention group will be required to obtain medical clearance from their general practitioner before starting the HIIT sessions. These sessions, led by a qualified exercise physiologist (the instructor) and adhering to standard protocols [39,40], will take place in the Aerobic Exercise Laboratory at Emory University's Rehabilitation Hospital. Using “spin cycle ergometers” (Schwinn), sessions will be conducted in small groups of 5 or fewer participants at specified times on Mondays, Wednesdays, and Fridays, spanning 8 weeks. Each participant will undergo a maximum of 24 HIIT sessions. Each session will consist of a 5-minute warm-up, followed by an interval-based workout phase with steady up-tempo cadences, sprints, climbs, and interspersed recovery periods. A 5-minute cooldown will conclude each session. The workout sessions will initially last 20 minutes and will progressively increase in time based on participants’ tolerance and instructor recommendations. Each session will include “active rest” periods where resistance is reduced to lower HR, alternating with high-intensity intervals featuring sprints or climbs to elevate HR. The duration of active rest versus high-intensity intervals will be adjusted according to individual responses and target HR. To monitor and maintain intensity within the target HR range, participants will wear Polar H10 chest strap HR sensors [41]. The target HR will be calculated using the Karvonen method [42]. Exercise intensity will begin at 75% of the estimated maximum HR reserve (HRR) and will increase by 5% every week, as tolerated or deemed necessary by the instructor, over the 8-week intervention period. During the workout phase, the target HRR reserve will be maintained by averaging increases and decreases in intensity or HR with a target to maintain within a 10% offset from the HRR goal [39,40]. Participants will need to adhere to within-session HR targets at an 80% rate (or greater) for a session to be counted as attended and participants will need to attend 19 out of 24 sessions to be included as a “completer” in the final data analysis. To date, our interventions have yielded a within-session adherence rate to the prescribed intervention of 91% (as measured by HR) and a retention rate of 85% [40,43-45]. Participants’ weight and body composition will be measured weekly.

To ensure high compliance in session attendance, the instructor will hold weekly one-on-one meetings with participants to provide personalized feedback and encouragement. Participants’ HR data will also be reviewed during these meetings. Additionally, the study coordinator will remind participants of their scheduled sessions 1 day in advance through phone calls or texts. Attendance in sessions will be closely monitored, and records of attended exercise sessions will be maintained. Participants who miss sessions will be contacted via phone calls to encourage attendance.

Any adverse events occurring during or after HIIT sessions will be documented, with medical advice sought if necessary. Both intervention and control participants will receive instructions to maintain a eucaloric diet throughout the study. Dietary adherence will be monitored biweekly by a registered dietitian using the Automated Self-Administered 24-Hour Dietary Assessment Tool (National Cancer Institute) [36]. This tool will be administered via phone calls 3 times a week, covering 2 weekdays and 1 weekend day. Additionally, control participants will be instructed to refrain from engaging in intense physical activities during the study period. Physical activity adherence will be assessed biweekly using the short form of IPAQ, also administered via phone calls [32].

### Procedures

The details about the measurements, study tools, and timelines are outlined in Table 1.

**Table 1:** Measurements, study tools, and study timeline.

Variables	Components	Study tools	Baseline		8 weeks
Study feasibility	Response rate, screening yield, enrollment rate, time to enrollment, intervention compliance, resource use, and retention rate	REDCap^a^ database	✓		✓
Intervention feasibility	Qualitative and quantitative research	Feasibility of Intervention Measure [46]	✓		✓
Intervention feasibility	Qualitative and quantitative research	In-depth interviews	✓		✓
Intervention acceptability	Qualitative and quantitative research	Theoretical Framework of Acceptability questionnaire [47]	✓		✓
Intervention acceptability	Qualitative and quantitative research	In-depth interviews	✓		✓
Intervention appropriateness	Qualitative and quantitative research	Intervention Appropriate Measure [46]	✓		✓
Intervention appropriateness	Qualitative and quantitative research	In-depth interviews	✓		✓
Participants’ expectations of and experiences with the intervention	Qualitative research	In-depth interviews	✓		✓
Sociodemographics	Age, sex, marital status, education, and occupation	WHO STEPS^b^ [34] and NHANES^c^ questionnaires [35]	✓		x^c^
Eligibility criteria	Inclusion and exclusion criteria^e^	Short-form IPAQ^f^ [32]	✓		x
Behavioral measures	Dietary habits	ASA24^g^ dietary assessment tool [36]	✓^h^	✓^h^	
Behavioral measures	Physical activity	Short-form IPAQ [32]	✓^i^	✓^i^	
Behavioral measures	Smoking	WHO STEPS [34] and NHANES questionnaires [35]	✓		✓
Behavioral measures	Alcohol consumption	WHO STEPS [34] and NHANES questionnaires [35]	✓		✓
Physical measures	Height	Stadiometer	✓		x
Physical measures	Weight	Digital weighing scale	✓		✓
Physical measures	Waist circumference	Inelastic measuring tape	✓		✓
Physical measures	Hip circumference	Inelastic measuring tape	✓		✓
Physical measures	BP^j^	DINAMAP BP apparatus	✓		✓
Physical measures	Body composition	Bioimpedance analysis	✓		✓
Biochemical measures	OGTT^k^ (0, 30, and 120 minutes)	Enzymatic assays	✓		✓
Biochemical measures	Insulin levels at 0 and 30 minutes	Immunoassays	✓		✓
CGM^k^	Proportion of time and mean time spent in nocturnal (00:00-06:00) normoglycemia (60 to <100 mg/dl) [48]	Dexcom G6 Pro (DexCom, Inc)	✓^l^	✓^l^	

^a^REDCap: Research Electronic Data Capture.

^b^WHO STEPS: World Health Organization STEPwise approach to noncommunicable disease risk factor surveillance.

^c^NHANES: National Health and Nutrition Examination Survey.

^d^x: the particular variable will not be collected during that particular timepoint.

^e^Inclusion criteria: aged 35-65 years, overweight or obese (body mass index ≥25 kg/m² [≥23 kg/m² if Asian descent]), physically inactive (less than 150 minutes per week of moderate-intensity physical activity and less than 75 minutes per week of vigorous-intensity physical activity and <600 metabolic equivalent task–minutes per week), and a diagnosis of isolated impaired fasting glucose (fasting plasma glucose 100-125 mg/dL and 2-hour plasma glucose<140 mg/dL). Exclusion criteria: diagnosis of diabetes, diagnosis of other chronic illnesses (eg, cardiovascular disease, stroke), current smoker, history of anemia, currently enrolled in weight loss programs, currently enrolled in any regular exercise programs, currently following a specific diet (eg, ketogenic diet, Mediterranean diet), currently taking weight-loss medications or drugs known to influence glucose tolerance (steroids and antipsychotics), currently taking beta-blockers or calcium channel blockers, underwent bariatric surgery or plans to undergo the surgery during the study period, plans to relocate outside the study area during the study period, pregnant, or breastfeeding.

^f^IPAQ: International Physical Activity Questionnaire.

^g^ASA24: Automated Self-Administered 24-Hour Dietary Assessment Tool.

^h^Dietary habits will be assessed biweekly throughout the study duration.

^i^Physical activity of control participants will be assessed biweekly throughout the study duration.

^j^BP: blood pressure.

^k^OGTT: oral glucose tolerance test.

^l^All participants will be fitted with the continuous glucose monitoring device 10 days before the intervention, and they will be trained to replace the device every 10 days until 10 days post intervention.

### Study Feasibility

Table 2 shows the study feasibility metrics. Continuous data collection on feasibility parameters, such as response rate, screening yield, enrollment rate, time to enrollment, intervention compliance, resource use (cost and staff time), and retention rate, will be conducted throughout the study.

**Table 2:** Study feasibility metrics.

Parameters	Calculations
Response rate	Number of individuals responded to the invitation/number of individuals invited
Screening yield	Number of individuals diagnosed with i-IFGa/number of individuals screened
Enrollment rate	Number of individuals enrolled/number of individuals diagnosed with i-IFG
Time to enrollment	Average time taken from sending the invitation to enrolling one participant in the trial
Intervention compliance	Number of HIITb sessions attended/Total number of HIIT sessions
Resource use	Program costs: Includes screening cost, cost of procedures, intervention cost, participant incentives, and other costs. Staff time: Time spent screening and recruiting participants, time spent delivering the intervention, time spent making phone calls to participants, time spent implementing the study procedures, and time spent on baseline and follow-up assessments.
Retention rate	Number of participants attended follow-up visits/number of participants enrolled

^a^i-IFG: isolated impaired fasting glucose.

^b^HIIT: high-intensity interval training.

### Intervention, Feasibility, Acceptability, and Appropriateness

The Feasibility of Intervention Measure (FIM) will evaluate the feasibility of the intervention, encompassing questions regarding its implementability, possibility, doability, and ease of use [46]. The acceptability of the intervention will be assessed through the Theoretical Framework of Acceptability (TFA) questionnaire, which explores affective attitude, burden, ethicality, perceived effectiveness, intervention coherence, self-efficacy, opportunity costs, and general acceptability [47]. The Intervention Appropriate Measure (IAM) will evaluate the appropriateness of the intervention, including questions about its fittingness, suitability, applicability, and alignment with participants’ needs [46]. Responses to the questions in all 3 questionnaires will be recorded on a Likert scale of 1 to 5. The mean total score for each of these scales will be calculated by combining the individual Likert points of each scale. Higher scores on the FIM, TFA, and IAM scales indicate greater feasibility, acceptability, and appropriateness, respectively, among participants.

### Continuous Glucose Monitoring

By providing 288 glucose measurements per day throughout the 8-week intervention and 10-day follow-up, continuous glucose monitoring (CGM) can track the dynamic changes in fasting glucose levels induced by HIIT [48]. This can help identify when the effects of HIIT on fasting glucose levels become evident and whether these effects are sustained after the intervention, which may not be captured by a single blood glucose measurement taken after 8 weeks. All participants, regardless of their assigned treatment, will be fitted with a CGM device on their abdominal area upon enrollment. The CGM device, Dexcom G6 Pro CGM system (DexCom, Inc), will be used in blinded mode to minimize bias and ensure that it does not influence the study outcomes. Participants will be instructed to eat their last meal by 10 PM daily after the CGM fitting. They will wear CGM devices for approximately 80 days, including 10 days prior to the intervention, the 8-week intervention period, and 10 days following the intervention. Participants will be trained on how to replace the device every 10 days, using the instructions provided in the manual [49], during the first study visit. The adequacy of CGM data will be evaluated using the following criteria: a minimum of 80% of the potential 288 glucose values per day should be present for any 7 consecutive days, commencing from the day following sensor insertion [50].

### Clinical Measures

Data on health behaviors, physical measurements, and biochemical measurements will be collected at both baseline and 8 weeks.

#### Health Behaviors

Physical activity levels will be assessed using the short form of IPAQ [32] and dietary intake with the Automated Self-Administered 24-Hour Dietary Assessment Tool questionnaire [36]. Data on smoking and alcohol use will be obtained using questions adapted from the WHO STEPS [34] and the National Health and Nutrition Examination Survey questionnaires [35].

#### Physical Measures

Physical measurements will be taken following standard protocols [37,51]. Height will be measured using a stadiometer (Welch Ally—Scale-Tronix) with an accuracy of 0.1 cm. Weight will be assessed using a digital weighing scale (Welch Ally—Scale-Tronix) with precision to the nearest 0.1 kg. Waist and hip circumferences will be measured using an inelastic measuring tape (BaumGartens) with a precision of 0.1 cm. Blood pressure will be measured using the DINAMAP automatic blood pressure apparatus (GE HealthCare). Body composition measures, including fat mass, muscle mass, fat-free mass, visceral adipose tissue mass, and fat percent, will be obtained using the bioimpedance analysis.

#### Biochemical Measures

Participants will undergo an OGTT following standard protocols [52,53]. The test will be conducted after an overnight fast of at least 10 hours, with the session scheduled between 7 and 9 AM. Venous blood samples will be collected at 0, 30, and 120 minutes after ingesting a 75-g oral glucose load dissolved in 250-300 mL of water, consumed over 5 minutes. Additionally, blood samples for insulin will be obtained at 0 and 30 minutes after glucose load ingestion. Blood samples will be processed and analyzed at the Emory Medical Laboratory (EML). EML is a fully accredited and licensed clinical laboratory, actively participating in the College of American Pathologists Laboratory Accreditation Program. Additionally, it holds Clinical Laboratory Improvement Amendments certification through the Centers for Medicare and Medicaid Services. EML is also duly licensed by the state of Georgia. Glucose levels will be assessed through enzymatic assays and insulin levels via immunoassays based on the EML protocol [53]. All these analyses will use kits provided by Beckman Coulter Inc and will be performed on a Beckman Coulter analyzer.

##### Indices of ß Cell Function and Insulin Resistance

Table 3 provides details on the indices of ß Cell function and insulin resistance derived from glucose and insulin levels. Early-phase insulin secretion will be assessed using the insulinogenic index [54], while total ß cell function with be evaluated with the oral disposition index [55] and homeostatic model assessment of ß cell function [56]. Whole-body insulin resistance will be determined using the Matsuda index [57] and homeostatic model assessment of insulin resistance [56], while tissue-specific insulin resistance will be assessed with the hepatic insulin resistance index [58] and muscle insulin sensitivity index [58].

**Table 3:** Indices of ß-cell function and insulin resistance.

ß cell function or IR^a^ and components		Indices	Formula
**ß cell function**			
	Early-phase insulin secretion:	IGI^b^ [54]	(I_30_^c^–I_0_^d^)/(G_30_^e^–G_0_^f^)
	ß cell function	DI_O_^g^ [55]	([ I_0-30_/ G_0-30_]×[1/I_0_]) I0 in µU/l G0 in mmol/l
	ß cell function	HOMA-B^h^ [56]	(20×I_0_)/(G_0_–3.5) I0 in µU/l G0 in mmol/l
**Insulin resistance**			
	Whole-body insulin sensitivity	Matsuda index [57]	10,000 /√((G_0_×I_0_)×(G_mean_^i^×I_mean_^j^))
	Insulin resistance	HOMA-IR^k^ [56]	(I_0_×G_0_)/22.5 I0 in µU/L G0 in mmol/l
	Hepatic insulin resistance	HIRI^l^ [58]	(G_0_–G_30_[AUC^m^]×I_0_-_30_[AUC]) G0 in mg/dl I0 in µU/ml
	Muscle insulin resistance	MISI^n^ [58]	(dG/dt^o^)/Ī^p^

^a^IR: insulin resistance.

^b^IGI: insulinogenic index.

^c^I_30_: mean insulin at 30 minutes during the oral glucose tolerance test.

^d^I_0_: mean insulin at 0 minutes during the oral glucose tolerance test.

^e^G_30_: mean glucose at 30 minutes during the oral glucose tolerance test.

^f^G_0_: mean glucose at 0 minutes during the oral glucose tolerance test.

^g^DI_O_: oral disposition index.

^h^HOMA-B: homeostatic model assessment of ß cell function.

^i^G_mean_: mean glucose during the 2-hour oral glucose tolerance test.

^j^I_mean_: mean insulin during the 2-hour oral glucose tolerance test.

^k^HOMA-IR: homeostatic model assessment of insulin resistance.

^l^HIRI: hepatic insulin resistance index.

^m^AUC: area under the curve during the first 30 minutes of the oral glucose tolerance test.

^n^MISI: muscle insulin sensitivity index.

^o^dG/dt: slope of the regression line from the peak of the plasma glucose curve to its nadir (mmol/L/min).

^p^Ī: mean insulin concentration over the 2-hour duration of the oral glucose tolerance test (pmol/L).

#### Qualitative Research

Participants assigned to the intervention group will be invited to take part in in-depth interviews both before and after the HIIT intervention. Trained interviewers will administer these interviews either in person during scheduled study visits or via Zoom within 1 week of the visits if participants are unable to attend in person. The interviews will be guided by interview guides specifically developed for the study and piloted with members of the study team. Preintervention interviews will delve into participants’ prediabetes history, physical activity and dietary habits, perceptions of body size and image, as well as their comfort levels, perceived difficulty, confidence, and expectations regarding the HIIT intervention. Post-intervention interviews will focus on participants' experiences with the intervention. Every effort will be made to interview both dropouts and active participants to ensure a comprehensive understanding of program acceptability and to identify barriers and facilitators to adherence. All interviews will be audio recorded, transcribed verbatim, and deidentified for analysis.

### Outcomes

#### Primary Outcomes

- Quantitative measures: (1) feasibility metrics and (2) mean FIM, TFA, and IAM scores.

Qualitative measures: participants’ experiences, perceptions, and satisfaction with the HIIT intervention, and facilitators and barriers to participation.

#### Secondary Outcomes

- Between-group differences in changes in the following parameters from baseline to 8 weeks: (1) mean FPG and insulin levels, (2) indices of β cell function and insulin resistance, and (3) weight, body composition, waist and hip circumferences, and blood pressure.
- CGM metrics: (1) between-group differences in the proportion of time and mean time spent in nocturnal (12 to 6 AM) [50] normoglycemia (60 to <100 mg/dL) during the 8-week intervention period and the 10 days following the intervention, and (2) within-participant differences in the proportion of time and mean time spent in nocturnal (12 and 6 AM) normoglycemia (60 to <100 mg/dL) between exercise and non-exercise days during the 8-week intervention period.

### Data Management

The study coordinator will enter questionnaire data, as well as physical and biochemical measurements directly into Emory University’s REDCap database [30]. This database will feature validation checks to ensure data accuracy, along with skip patterns facilitated by branching logic functions. The principal investigator (ST) will constantly review the data for any errors, promptly flagging any errors for correction by the study coordinator. Upon completion of data entry and cleaning, a master copy of the data set will be generated and securely stored within the REDCap database. CGM raw data (in CSV file format per participant) will be downloaded from the DexCom Clarity software and uploaded to REDCap. Access to these datasets will be limited to the study coordinator and the principal investigator for confidentiality and data security purposes.

### Sample Size Calculation

Assuming a Cohen *d* of 0.3 to <0.7 (medium standardized effect size) [25,59] for FPG in the planned main trial, with an alpha of 5% and a power of 90%, a sample size of 15 participants per treatment group is deemed necessary for this pilot study. Factoring in a 10% loss to follow-up in each group, the total sample size was estimated to be 34 participants (17 per group).

### Statistical Analysis

#### Quantitative Research

##### Objective 1

Continuous variables will be summarized using either mean (SD) or median (IQR), depending on their distribution, which will be visually assessed through histograms. Categorical variables will be presented as counts (n) and percentages (%).

##### Objective 2

The analyses will adhere to the “intention-to-treat” principle. Between-group differences in changes in continuous variables from baseline to 8 weeks will be analyzed using mixed-effects linear regression models, while categorical variables will be assessed with log-binomial models. Skewed variables will be log-transformed prior to analysis. Fixed effects will include the study group (intervention vs control), timepoint (follow-up vs baseline), and the interaction between the study group and timepoint. Random effects will be specified for participants to account for the correlation between repeated measurements on the same individual. The *P* value for the study group-by-timepoint interaction will be used to evaluate the difference in changes between the study groups. The correlation between changes in fasting glucose levels and the indices from baseline to 8 weeks will be assessed using either Pearson or Spearman correlation coefficients, depending on the nature of the data distribution. Mixed-effects linear regression models will be used to compare CGM metrics between study groups, adjusting for baseline values. These models will also examine within-participant differences in CGM metrics between exercise and non-exercise days. Statistical significance will be considered with a 2-sided *P* value<.05 with no adjustments for the multiplicity of comparisons. All analyses will be conducted using Stata (version 18.0; StataCorp LLC).

#### Qualitative Research

In-depth interviews will be conducted with intervention group participants and dropouts. All interviews will be audio recorded and transcribed verbatim for analysis. The qualitative analysis plan involves 2 main components: a framework-driven analysis of intervention acceptability data and a thematic analysis focusing on participant expectations, experiences, barriers, and facilitators in undergoing the HIIT intervention. For the framework-driven analysis, a deductive codebook containing the TFA dimensions will be applied to both baseline and postintervention interview data. This approach aims to provide a comprehensive and longitudinal understanding of HIIT acceptability among program users, comparing results across timepoints and between those who remained in the program and study dropouts. Additionally, an inductive approach will be used to create a codebook of inductive codes around other aspects of acceptability, program barriers, facilitators, experiences, and sustainability through a close reading of the transcripts. Once the data is coded, thick descriptions of individual codes will be developed, including structured comparisons such as between baseline and postintervention interviews, program adherents and dropouts, men and women, and older and younger participants. These comparisons will guide data reporting and program adaptation for further trials, providing insights into the diverse experiences and perspectives of participants.

### Challenges and Mitigation Strategies

The potential challenges that could be encountered at various stages of the study and the corresponding mitigation strategies are outlined in Table 4.

**Table 4:** Potential challenges and proposed mitigation strategies.

Study stage	Challenges	Mitigation strategies
Identifying potential participants	Insufficient number of potentially eligible individuals	We will use physician referrals as an additional recruitment strategy.
Screening	Low yield of screening	The 3-step screening procedure was carefully designed, drawing upon insights from our previous research and existing literature, to target individuals who are likely to have i-IFG^a^.
Intervention	Low HIIT^b^ compliance	The study coordinator will remind participants of their scheduled exercise sessions through phone calls. Additionally, the coordinator will regularly review the attendance log, providing motivation and support to participants with low attendance levels. The exercise instructor will hold weekly one-on-one meetings with participants to review their progress and provide motivation, specifically targeting those with low attendance levels.
Procedures	Periodic data gaps with CGM^c^ whenever the receiver is located more than 5 feet	The CGM data will be assessed for adequacy based on the following criteria: Data points must be present for at least 80% of the possible 288 glucose values per day for any 7 consecutive days, starting on the day after sensor insertion.
Follow-up	Low retention rate	Compensation for time and parking: (1) participants will receive a US $50 gift card upon completion of the study and (2) parking fees at study sites will be covered. Building rapport: study staff will create a warm and supportive environment during study visits, fostering a sense of trust and comfort. Ongoing support: the study coordinator will provide continuous support through regular phone calls. This proactive approach ensures that participants feel connected to the study outside of scheduled visits. The study coordinator will address any concerns, answer queries, and offer encouragement, reinforcing a sense of partnership between participants and the research team.

^a^i-IFG: isolated impaired fasting glucose.

^b^HIIT: high-intensity interval training.

^c^CGM: continuous glucose monitoring.

### Ethical Considerations

The study protocol was approved by the institutional review board of Emory University, Atlanta, USA (MOD001-STUDY00005855). All participants will provide written informed consent prior to study participation. Participant identifiers will be kept strictly confidential in a secure REDCap database, accessible only by the principal investigator and study coordinator. Data will be deidentified before analysis. Participants will receive a US $50 gift card as compensation for their participation.

## Results

Table 5 shows the study timeline. Recruitment for the study is scheduled to begin in February 2025, with follow-up expected to be completed by the end of September 2025. We plan to publish the study findings by the end of 2025.

**Table 5:** Study timeline^a^.

	2023		2024					2025			
	May	July-December	January-March	April-June	July-September	October-December	January-March		April-June	July-September	October-December
Received funding	✓										
Finalizing the study protocol and study tools		✓									
Clinical trial registration		✓									
Obtaining approvals: NIH^b^, NCATS^c^, and IRB^d^			✓	✓	✓						
REDCap database setup						✓					
Screening and recruitment							✓		✓		
Baseline assessments							✓		✓		
Baseline in-depth interviews							✓		✓		
HIIT^e^ intervention							✓		✓		
8-week assessments										✓	
In-depth interviews at 8 weeks										✓	
Data entry							✓		✓	✓	
Data analysis										✓	✓
Study report and publications											✓
Conferences and scientific meetings											✓

^a^The cells colored in gray showing the timeline for various activities.

^b^NIH: National Institutes of Health.

^c^NCATS: National Center for Advancing Translational Sciences.

^d^IRB: institutional review board.

^e^HIIT: high-intensity interval training.

## Discussion

### Expected Findings

This proof-of-concept study will generate data on the feasibility and acceptability of the HIIT intervention, as well as participants’ experiences and satisfaction levels. Additionally, the study will offer preliminary estimates on the efficacy of HIIT in reducing FPG levels and addressing the pathophysiology of i-IFG.

### Strengths and Limitations

To our knowledge, this study will be the first to assess the feasibility and acceptability of a HIIT intervention exclusively among individuals with i-IFG. Additionally, we adhered to the PRISMA-P (Preferred Reporting Items for Systematic Review and Meta-Analysis Protocols) statement [60] when reporting the details of this study protocol. However, there are some limitations. We will assess the pathophysiological abnormalities in i-IFG using indices derived from the OGTT and fasting insulin levels instead of gold-standard methods like the intravenous glucose tolerance test and glycemic clamps [61-63]. However, these indices have demonstrated strong correlations with estimates obtained from the gold-standard methods [61-63]. Additionally, our reliance on a single OGTT may be subject to day-to-day variability in glucose tolerance status. Nevertheless, strict adherence to standardized protocols for conducting the OGTT [52,53] should help minimize this variability to a significant extent.

### Conclusions

The results of this study are expected to guide the design and implementation of an RCT to assess the efficacy of HIIT intervention in reducing diabetes incidence and achieving remission in individuals with i-IFG.

## Abbreviations

CGM: continuous glucose monitoring
CONSORT: Consolidated Standards of Reporting Trials
EML: Emory Medical Laboratory
FIM: Feasibility of Intervention Measure
FPG: fasting plasma glucose
GCRC: Georgia Clinical Research Center
HbA1c: hemoglobin A1c
HIIT: high-intensity interval training
HR: heart rate
HRR: heart rate reserve
i-IFG: isolated impaired fasting glucose
i-IGT: isolated impaired glucose tolerance
IAM: Intervention Appropriate Measure
IPAQ: International Physical Activity Questionnaire
OGTT: oral glucose tolerance test
PRISMA-P: Preferred Reporting Items for Systematic Review and Meta-Analysis Protocols
RCT: randomized controlled trial
REDCap: Research Electronic Data Capture
STEPS: STEPwise approach to noncommunicable disease risk factor surveillance
TFA: Theoretical Framework of Acceptability
